# A genomic Neolithic time transect of hunter-farmer admixture in central Poland

**DOI:** 10.1038/s41598-018-33067-w

**Published:** 2018-10-05

**Authors:** D. M. Fernandes, D. Strapagiel, P. Borówka, B. Marciniak, E. Żądzińska, K. Sirak, V. Siska, R. Grygiel, J. Carlsson, A. Manica, W. Lorkiewicz, R. Pinhasi

**Affiliations:** 1 Department of Evolutionary Anthropology, University of Vienna, Vienna, Austria; 2 School of Archaeology, and Earth Institute, University College Dublin, Dublin, Ireland; 3 CIAS, Department of Life Sciences, University of Coimbra, Coimbra, Portugal; 4 Biobank Lab, Department of Molecular Biophysics, Faculty of Biology and Environmental Protection, University of Lodz, Lodz, Poland; 5 BBMRI.pl Consortium, Wrocław, Poland; 6 Department of Anthropology, Faculty of Biology and Environmental Protection, University of Lodz, Lodz, Poland; 7 Department of Anthropology, Emory University, Atlanta, United States of America; 8 Department of Zoology, University of Cambridge, Cambridge, United Kingdom; 9 Museum of Archaeology and Ethnography in Lodz, Lodz, Poland; 10 Area 52 Research Group, School of Biology and Environment Science/Earth Institute, University College Dublin, Dublin, Ireland

## Abstract

Ancient DNA genome-wide analyses of Neolithic individuals from central and southern Europe indicate an overall population turnover pattern in which migrating farmers from Anatolia and the Near East largely replaced autochthonous Mesolithic hunter-gatherers. However, the genetic history of the Neolithic transition in areas lying north of the European Neolithic core region involved different levels of admixture with hunter-gatherers. Here we analyse genome-wide data of 17 individuals spanning from the Middle Neolithic to the Early Bronze Age (4300-1900 BCE) in order to assess the Neolithic transition in north-central Poland, and the local impacts of hunter-farmer contacts and Late Neolithic steppe migrations. We evaluate the influence of these on local populations and assess if and how they change through time, reporting evidence of recurrent hunter-farmer admixture over three millennia, and the co-existence of unadmixed hunter-gatherers as late as 4300 BCE. During the Late Neolithic we report the appearance of steppe ancestry, but on a lesser scale than previously described for other central European regions, with evidence of stronger affinities to hunter-gatherers than to steppe pastoralists. These results help understand the Neolithic palaeogenomics of another central European area, Kuyavia, and highlight the complexity of population interactions during those times.

## Introduction

The knowledge about the genetic history of prehistoric Europeans has increased substantially during the past three years following a series of studies that have provided new insights about the ancestry and affinities of Neolithic, Chalcolithic and Bronze Age populations from various parts of Europe^1–19^. Recent ancient DNA (aDNA) genome-wide studies have particularly addressed the extent of genetic admixture between Neolithic farmers and hunter-gatherers (HG) and found evidence of regional variability in the level and nature of the admixture in Iberia^5,9,19,20^, south-east^6,10,13,16^, central^1,2,7,15^, north-eastern^4,6,14^, and northwestern Europe^5,8^. In regions such as the Baltic, however, genomic data has shown that hunter-gatherer populations adopted farming without a significant genetic input from farming populations^14^. In light of these results, north-central Europe is particularly important to understand the genetic history of prehistoric Europeans. Here, the line of maximum north and north-eastern reach of settlement of Neolithic farmers of Linear Pottery culture (LBK) and post-Linear Pottery cultures (post-LBK) ran through the North European Plain. These farmers represented the first stage of neolithization of this part of Europe and they were allochthonous populations whose ancestry can be traced back to Anatolian early farmers, with only minor admixture of European hunter-gatherers obtained in south-eastern Europe and the Carpathian Basin^1,12,16^. In the territories like north-central Poland these farmers came into contact with well established hunter-gatherers communities of the Baltic region, which in turn occupied habitats not attractive for the agricultural lifestyle of LBK and post-LBK cultures. As a consequence, a borderland between the Neolithic farmer and HG worlds was constituted where farmers and HGs coexisted with each other for over one thousand years (during the fifth millennium BCE)^21–23^.

The Kuyavia region is one of the most representative areas of north-central Poland from the point of view of these processes when considering the archaeological record. Farming was introduced to this region with the arrival of LBK farmers ca. 5400 BCE^24–26^. During the 5^th^ millennium BCE, following the dissolution of the LBK, Kuyavia continues to be populated by post-Linear Pottery Neolithic cultural units of local expression such as the Stroke Band Pottery and later the Middle Neolithic Brześć Kujawski Group of the Lengyel culture (BKG)^24,27^. This cultural tradition is also known as the ‘Danubian Neolithic’ for its clear cultural ties with Carpathian Basin cultures on the one hand, and contrast to subsequent indigenous cultures on the other. After this phase, Kuyavia went through cultural transformations dominated by pan-European Middle and Late Neolithic cultural units like Funnel Beaker culture (TRB), Globular Amphora culture (GAC) and Corded Ware culture (CWC).

Around 4000 BCE, the Trichterbecherkultur, or Funnel Beaker culture^28^, appears, and becomes the main culture associated with the Neolithic expansion across Scandinavia and Atlantic Europe. This culture became responsible both for the final neolithization of territories like Poland, and for a further spread of agriculture to northern Europe. It has been suggested that the TRB communities were formed by indigenous northern European Mesolithic people who adopted farming locally rather than by incoming exogenous Danubian farmers from central Europe^28,29^. This model is supported by archaeological data which points to cultural, social, economic, and ideological transformations^28,29^. However, nuclear aDNA studies show that Scandinavian TRB farmers are genetically similar to today’s southern Europeans (particularly Sardinians), like other Early Neolithic farmers from central Europe^6,30^, albeit with the appearance of hunter-gatherer mitochondrial DNA haplogroups^31–34^. The results raise interesting questions about how and when did this admixture occur. Was it a short-term phenomenon that occurred at the end of LBK and post-LBK cultural tradition and reflected a disruption of the current cultural system and a massive transition of local HGs to agriculture; or rather was it a long term process lasting several hundred years within post-LBK cultural units like Rössen, Lengyel, or the Brześć Kujawski group in Poland? However, these specific questions cannot be directly addressed based on the current data as they require a much larger number of individuals from the various Central and Northern European post-LBK Middle Neolithic cultures.

By around 3100 BCE, the TRB culture was replaced by the Globular Amphora Culture across most of central and eastern Europe. This involved an economic transition marked by an increase in elements of pastoralism as well as farming^29^. The ancestry of the GAC had been disputed but recent data shows an absence of steppe ancestry in this culture^12,15^. The Corded Ware Culture, which have had a major input from migrating steppe pastoralists^2,3,9^, appears in the same region by the early part of the 3^rd^ millennium BCE.

The Kuyavia region is particularly useful for a population history analysis of the Polish lowlands for a number of reasons: (1) it was one of its most intensively settled regions during the entire Neolithic period and all major cultural units characteristic of central Europe are represented within it; (2) it has one of the best archaeological records of the Neolithic settlement in Poland, including well preserved human skeletons; and (3) it was situated on the north and north-east edge of the ‘farmers world’ during the Early and Middle Neolithic being particularly exposed to contacts with HGs.

**Figure 1:**
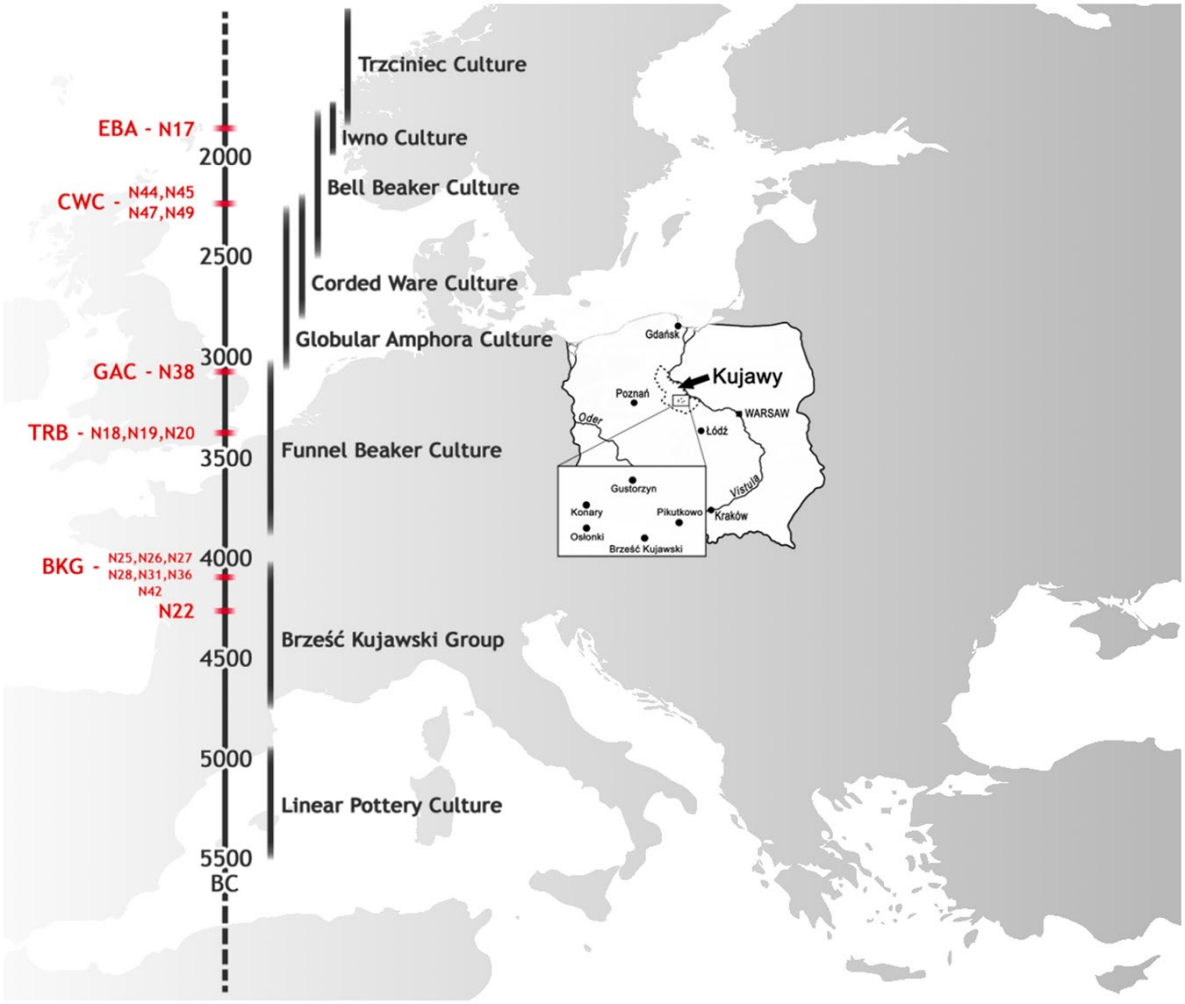
Timeline with culture acronyms and sample names, and map with location of archaeological sites.

Here we therefore investigate the population affinities, changes in genetic structure, and admixture, among Neolithic, Eneolithic, and Bronze Age populations from the Kuyavia region, north-central Poland. We report the results of a genomic time series of 17 individuals, spanning from the Middle Neolithic to the Early Bronze Age (4300-1900 BCE) (Fig. 1), with a particular focus placed on charting and interpreting intra-regional patterns of changes in genetic structure associated with the relevant sequence of archaeological cultures and the varying patterns of hunter-farmer admixture.

## Results

### Population affinities and hunter-farmer admixture patterns

**Figure 2:**
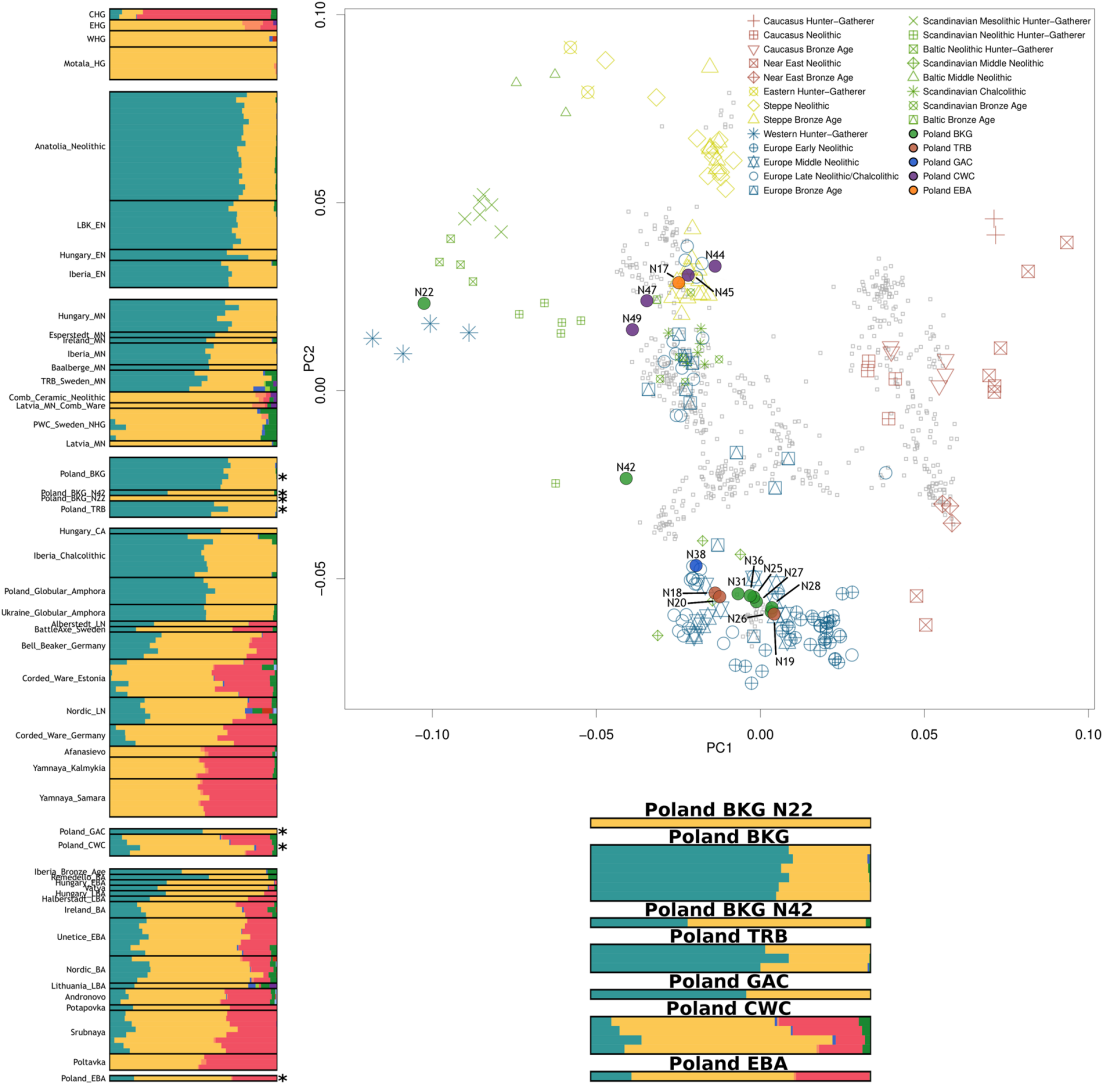
Principal component analysis with modern populations greyed out on the background (top-right), ADMIXTURE results with K = 10 with asterisks indicating the samples from this study (left) and those same samples amplified (bottom).

We obtained genome-wide data with mean genomic coverages between 0.71–2.50X (Supplementary Table S1). We first characterised the samples by comparing them to other ancient individuals using a principal component analysis (PCA) loaded on modern populations. With the exclusion of two outlier BKG individuals (N22 and N42), the Middle Neolithic Polish populations BKG and TRB from the Kuyavia region fall within the Early and Middle Neolithic cluster containing the LBK, Anatolian, Hungarian, and Iberian farmers (Fig. 2). Of the two BGK outliers, N22 clusters together with Western Hunter-Gatherer (WHG) individuals, whereas N42 appears in an intermediate position between the Early/Middle Neolithic and WHG clusters. During the Late Neolithic, two archaeological cultures coexisted in the Kuyavia region: the GAC and the CWC. The GAC individual, dated to the early occupation period of this culture (ca. 3000 BCE), is positioned close to the large cluster that includes Early/Middle Neolithic. The CWC individuals, dated to the late phase of the Corded Ware Culture (ca. 2250 BCE), which is approximately 750 years after the date of the GAC individual, cluster with other Eneolithic and Bronze Age individuals of central, north, and eastern European origin. The Early Bronze Age (EBA) individual is also positioned in this section of the PCA plot.

**Figure 3:**
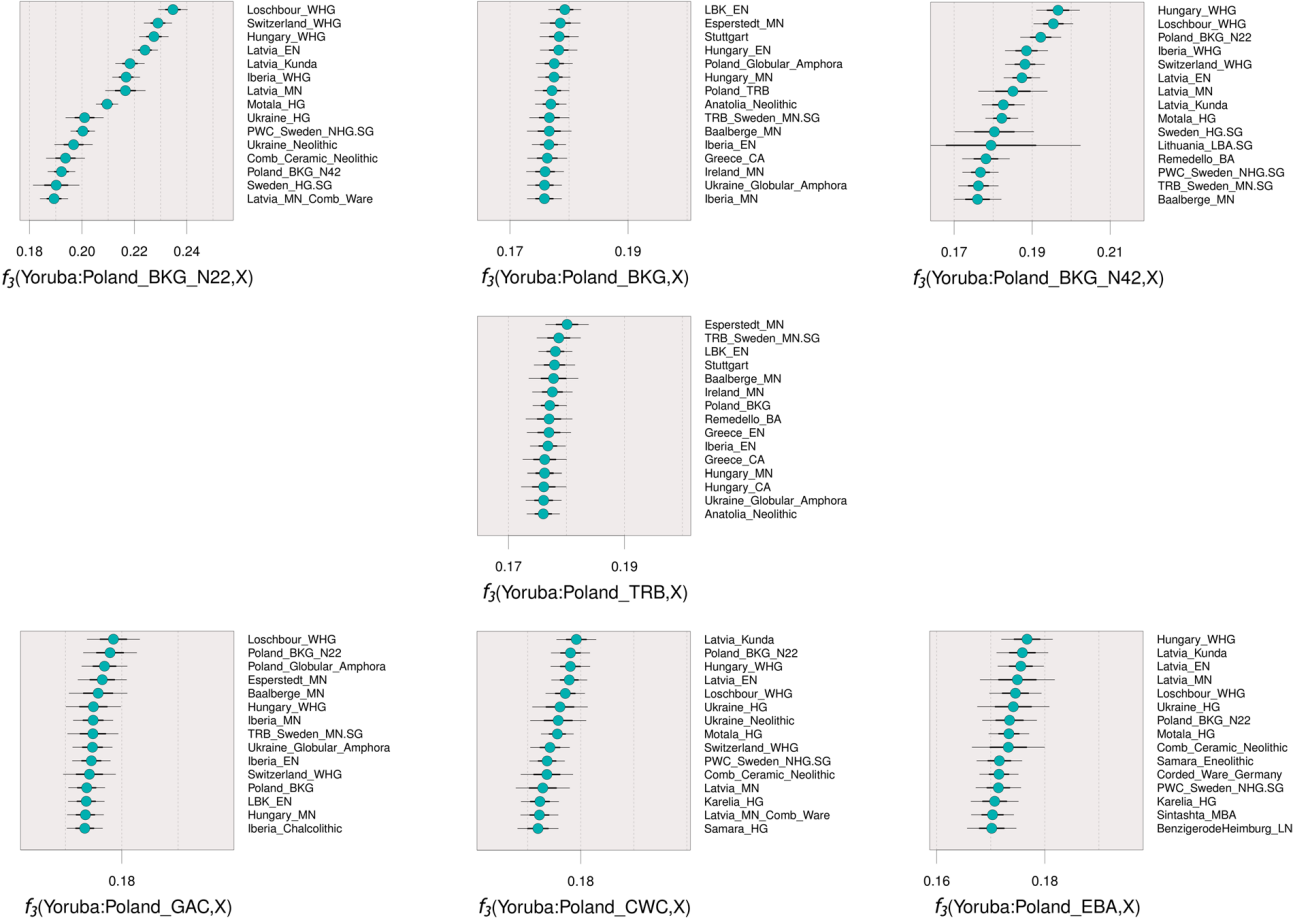
Top 15 outgroup *f*_3_ results for each culture and outlier. Thin and thick bars represent 1 and 3 standard deviations, respectively.

Next, we investigated the affinities and admixture patterns of the populations by applying an unsupervised model-based ancestry analysis using ADMIXTURE and *f*-statistics tests. The BKG (excluding the two outliers) appear on ADMIXTURE as mostly composed of the same genetic component present among Anatolian and LBK Early Neolithic farmers, and with a small HG component (Fig. 2). The results of outgroup-*f*_3_ tests confirm a major farmer ancestry, and hence that the BKG individuals in this region share most of their genetic drift with other Early and Middle Neolithic populations from central Europe (Fig. 3). We then assessed the affinities of the two outlier BKG individuals applying ADMIXTURE analysis (Fig. 2) and then comparing the results in relation to their mitochondrial haplogroups. Both N22 and N42 are females with Mesolithic mitochondrial DNA lineages U5a and U5b, respectively (Supplementary Tables S1–3). N22 was assigned to the same ancestral component that is found in all WHG, which is in agreement with its position with various WHG individuals in the PCA plot. Closer affinities to geographically proximal WHG from Luxembourg, Hungary, Switzerland, and Latvia than other HG are demonstrated by the results of outgroup-*f*_3_ and *f*_4_ tests (Fig. 3, Supplementary Table S4A). These make N22 the most recent individual (~4300 BCE) with a complete genomic WHG attribution to be found to date in an area occupied by Danubian Neolithic farmers. N42 was characterised as two-thirds WHG, and the remaining third assigned to the ADMIXTURE component characteristic of early north-west Anatolian farmers, in line with its intermediate position between the WHG cluster and Anatolian farmers on the PCA plot. These observations were supported by outgroup *f*_3_ tests of the form *f*_3_(Yoruba; *X*, *Y*), with *X* as N22 (Poland_BKG_N22) or N42 (Poland_BKG_N42), and *Y* as one of the published Early/Middle Neolithic and Mesolithic hunter-gatherer populations present in the merged dataset (Fig. 3). The highest levels of shared genetic drift were with WHG populations from Hungary, Luxembourg, Poland (N22), Iberia, Switzerland, and Latvia (Fig. 3, Supplementary Table S4A). Next we applied *f*_4_-statistics of the form *f*_4_(Yoruba, Poland_BKG (excluding N22&N42); Poland_BKG_N22, Loschbour_WHG/Hungary_WHG), and detected that N22 forms a clade with WHG from Luxembourg and Hungary to the exclusion of the BKG individuals from the same archaeological context (−0.420 < Z < −0.136) (Supplementary Table S4B). For N42, we also were not able to reject the null hypothesis that it forms a clade with these WHG [*f*_4_(Yoruba, Poland_BKG (excluding N22&N42); Poland_BKG_N42, Loschbour_WHG/Hungary_WHG), −2.687 < Z < −2.321]. These results are closer to the significance threshold (Z < 3), which is expected given the admixture of N42 with farmer populations, as shown in other analyses (Supplementary Table S4C). We tried to find an admixture date for N42 with ALDER^20,35^, following the approach applied by Lipson and colleagues^20^, but one of the helper individuals’ test run failed to fit. Since both outliers are female, we also investigated the possibility of sex-biased admixture in N42 using outgroup-*f*_3_ [*f*_3_(Yoruba; Poland_BKG_N42X, Anatolia_Neolithic/Loschbour_WHG)] and *f*_4_-statistics tests [*f*_4_(Yoruba, Poland_BKG_N42X; Anatolia_Neoltihic, Loschbour_WHG)] applied to X chromosome SNPs. Both analysis produced similar results showing that on the X chromosome N42 is symmetrically related to Anatolian Neolithic farmers and to Loschbour_WHG (Table S4C), although we caution that only between 1626 and 2085 SNPs were used.

We then analysed the TRB individuals. Results of ADMIXTURE and outgroup-*f*_3_ analysis, indicate that the TRB individuals shared a similar genetic composition as that of the BKG individuals (excluding the two outliers), but with a slightly higher HG component (Figs 2 and 3). Next we assessed how the Polish TRB individuals were related to the ones attributed to the same culture in Scandinavia^6^, responsible for the introduction of agriculture in that region, using the test *f*_4_(Yoruba, Poland_TRB; *X*, TRB_Sweden_MN) with *X* as Early and Middle Neolithic populations from northern Europe. Results indicate that the Polish TRB were as symmetrically related to the Scandinavian TRB as to other Middle Neolithic central European populations from Poland, Germany, and Hungary (0.287 < Z < 2.025) (Supplementary Table S4D). This result contrasts with those for other Early and Middle Neolithic individuals from Scandinavia and the Baltic who have hunter-gatherer affinities, with the Polish TRB being significantly closer to the Scandinavian TRB than to the Baltic Early and Middle Neolithic (3.764 < Z < 10.234) (Figs 2 and 3, Supplementary Table S4D).

**Figure 4:**
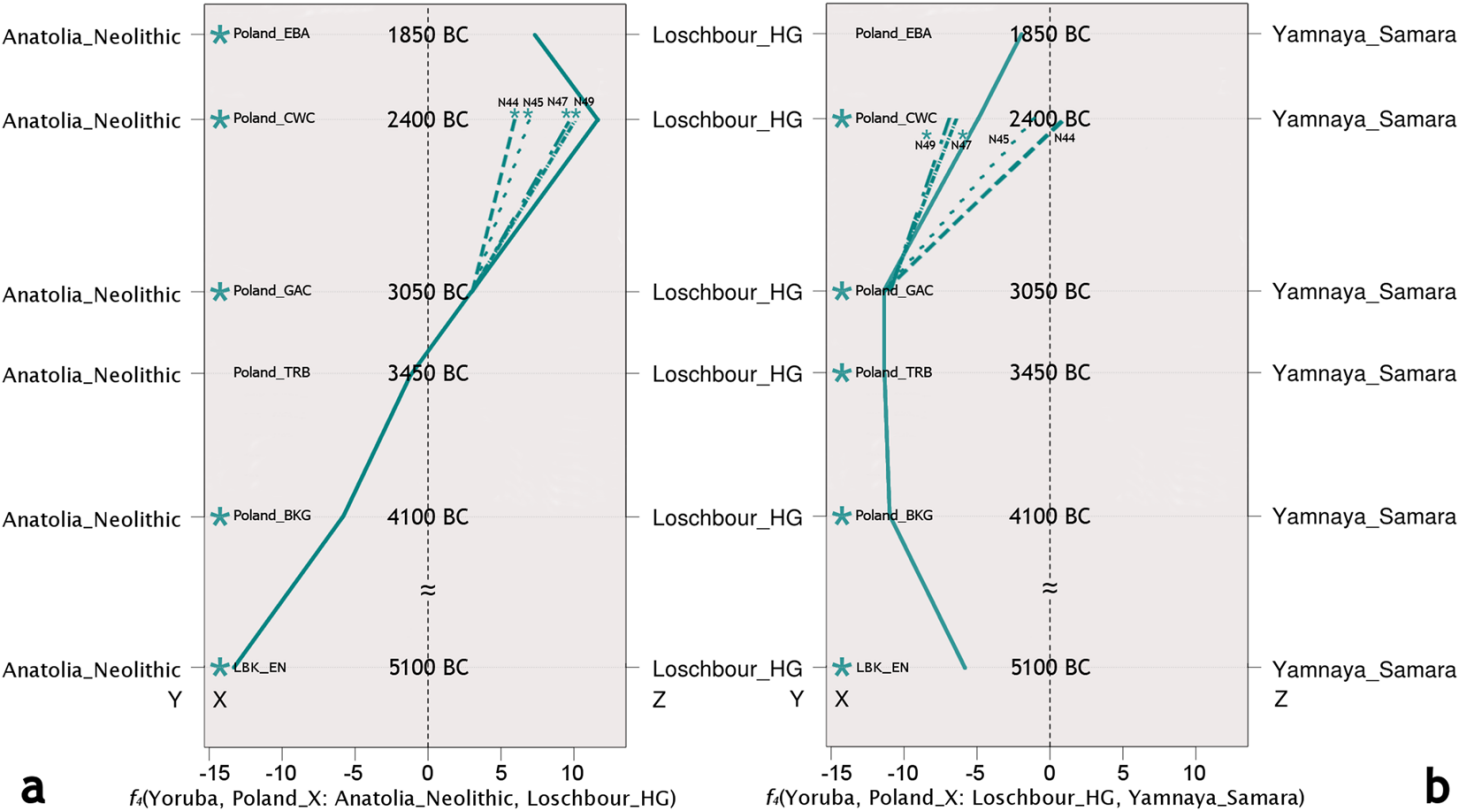
Chronologically ordered WHG (**a**) and steppe (**b**) ancestry variation along the time series. Z scores are presented instead of *f*_4_-statistic, for easier understanding of significance. Asterisks denote statistical significance (|Z| > 3).

**Figure 5:**
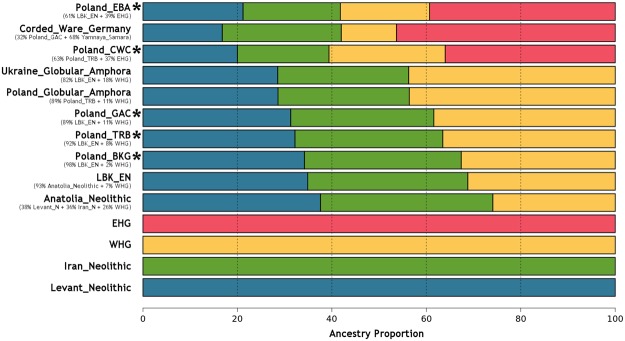
Ancestry proportions based on *qpAdm*. Visual representation of the main results presented in Supplementary Table S5. Populations from this study marked with an asterisk. Values and populations in brackets show the nested model results marked in green in Supplementary Table S5.

The observed pattern in ADMIXTURE of an increased component of HG ancestry in Poland’s Middle Neolithic compared to the LBK, led us to investigate whether admixture between farmers and hunter-gatherers only occurred in the earlier parts of the Neolithic in the Kuyavia region, or whether it continued throughout this period. ADMIXTURE ratios (Fig. 2) and *f*_4_-statistic tests of the form *f*_4_(Yoruba, *X*: LBK_EN, Loschbour_WHG), with *X* as the groups in the time series (Fig. 4a, Supplementary Table S4), show an increase over time in hunter-gatherer component from the BKG (ca. 4100 BCE) until the Late Neolithic (ca. 2250 BCE), suggesting some admixture events between hunter-gatherers and farmers in the Kuyavia region that at the same time remained genetically differentiated, either by hunter-gatherer groups inhabiting the same or nearby region, or by the influx of hunter-gatherer groups from more distant regions. Specifically, according to the test *f*_4_(Yoruba, Poland_TRB; LBK_EN, Loschbour_WHG) (Z = −2.302) by the end of the Middle Neolithic, the TRB individuals, dated to ca. 3400 BCE, shared as much genetic drift with hunter-gatherers as with Early Neolithic farmers (Fig. 4a, Supplementary Table S4D). The farmer-HG admixing process is also apparent ~400 years later, around 3000 BCE, in the Late Neolithic individual from the early GAC which shares the most genetic drift with both farmers and HG, according to outgroup *f*_3_ tests (Fig. 3). We further tested this with *qpWave/qpAdm* by modelling each of the groups as composed by four different ancestries basal to European populations: Levant Neolithic farmers, Iranian Neolithic farmers, Western Hunter-Gatherers, and Eastern Hunter-Gatherers (Fig. 5, Supplementary Table S5). This software compares a set of test populations against a set of outgroups in order to estimate mixture proportions and streams of ancestry. The results show a constant increase in the average amounts of WHG ancestry up to the GAC period, and the appearance of eastern hunter-gatherer (EHG) ancestry related to the expansion or Pontic steppe cultures by the CWC period (Fig. 5, Supplementary Table S5). The Polish and Ukrainian GAC individuals from^12,15^ seem to show even higher WHG ancestry than the individual from our study, but the test *f*_4_(Yoruba, Loschbour_WHG; Poland_GAC, Poland/Ukraine_Globular_Amphora)(Z = 1.143, −0.067, respectively), suggests those differences are not statistically significant (Supplementary Table S4E).

### The arrival of steppe groups

In the past, the Globular Amphora Culture has been regarded as one of first Indo-European cultures to precede and to be contemporaneous with the Corded Ware Culture^36^. While the CWC show evidence of steppe ancestry, as seen by the appearance of a Caucasus Hunter-Gatherer and Iranian Neolithic component in ADMIXTURE, the same analysis and *qpAdm* (Figs 2 and 5) indicate that it was absent in the GAC individual, and therefore suggest a non-steppe ancestry to this specific GAC individual. This is further supported by *qpAdm* tests that confirm that the GAC need not include steppe ancestry to explain her ancestry (Fig. 5, Supplementary Table S5) - a pattern also identified in the GAC individuals from a different site in Poland analysed by Tassi and colleagues^15^ (Supplementary Table S5).

The CWC individuals analysed here were part of a multiple grave that consisted of a total of 14 individuals. The archaeological study of the burials suggested that they were contemporaneous and possibly related^37^. This hypothesis was confirmed by kinship analysis of the N44, N45, N47, and N49 individuals, which shows orders of relatedness varying from second down to third order or unrelated (Supplementary Table S6). For the dyads where the coefficients show unrelated kinship (N45-N47 and N45-N49), those individuals are nevertheless always related to some other CWC individual. Two pairs of individuals with genetic similarities were observed on the PCA: N44:N45 and N47:N49 (Fig. 2, Supplementary Table S1) and therefore suggests that these form two separate clusters. We further examined the model of two separate clusters with *f*_4_-statistics of the form *f*_4_(Yoruba, Poland_CWC; Loschbour_WHG, Yamnaya_Samara) that show different patterns of affinities with hunter-gatherers and steppe populations (Fig. 4b, Supplementary Table S4F). A statistically significant closer relatedness with WHG than with the steppe seems to exist for N47:N49 (Z = −6.491 and Z = −6.379,respectively), but not for N44:N45 (Z = 0.505 and Z = −0.871, respectively) (Fig. 4b, Supplementary Table S4F). Some evidence of the differences between these pairs is also provided by mortuary context: all individuals in the burial were arranged in double or triple groups which most probably reflected the closer kinship of the individuals within them. The mortuary context therefore is in accord with the genetic results and indicate that the N44:N45 and N47:N49 dyads were each part of different burial groups. When compared to previously published CWC data^2,9^, our CWC group (not individuals) is genetically significantly closer to WHG than to steppe individuals (Z = −4.898), a result which is in contrast with those for CWC from Germany (Z = 2.336), Estonia (Z = 0.555), and Latvia (Z = 1.553) (Table 1). When applying *f*_4_-statistics of the form *f*_4_(Yoruba, Poland_CWC: Loschbour_WHG, Corded_Ware_Germany/Estonia/Latvia), the Polish CWC are statistically more similar to WHG than to other central/northern European CWC groups (Z = −4.009 when compared to Corded_Ware_Germany, and Z = −6.347 when compared to Corded_Ware_Estonia), but symmetrically related to WHG and Corded_Ware_Latvia (Z = −2.859) due to their higher HG ancestry (Table 1). Next, we tested whether the HG ancestry of the CWC individuals could have originated in Baltic populations by applying the test: *f*_4_(Yoruba, Poland_CWC; Latvia_EN, Loschbour_WHG/Poland_BKG_N22) (Supplementary Table S4F). The results reject the premise by producing symmetrical results (Z = −0.592, 0.222, respectively). To obtain further insight into the structure of the CWC group we tried to model it as the result of admixture between the GAC, WHG, and EHG/Yamnaya. The results show that they can be modeled with the GAC and EHG, but the model loses significance when the Yamnaya are used as the steppe source, from p = 0.168 to p = 0.016 (Supplementary Table S5). We were able to obtain significant models (p > 0.05) with the Yamnaya by analysing the two CWC dyads separately, with the results suggesting that while the N44:45 dyad can be modeled as a result of admixture between the GAC and the Yamnaya (p = 0.340), the N47:49 dyad requires also a small contribution from a WHG source (0.596 < p < 0.765) (Supplementary Table S5). The German Corded Ware, like the N44:N45 dyad, can be modeled without a WHG source (Supplementary Table S5). The Latvian Corded Ware were best modelled as admixture between local Neolithic populations with HG affinities and steppe migrants, with a complete absence of farmer ancestry^14^. These results indicate genetic substructure among the Corded Ware groups of central Europe.

**Table 1:** Comparative *f*_4_-statistics for Polish, German, Estonian, and Latvian Corded Ware. Values in bold denote statistical significance (|Z| > 3).

W	X	Y	Z	*f*_*4*_-stat	Z	SNPs
**Yoruba**	**Poland_CWC**	**LBK_EN**	**Loschbour_WHG**	**0.003629**	**10.416**	**568525**
**Yoruba**	**Corded_Ware_Germany**	**LBK_EN**	**Loschbour_WHG**	**0.001788**	**5.174**	**562932**
**Yoruba**	**Corded_Ware_Estonia**	**LBK_EN**	**Loschbour_WHG**	**0.002231**	**6.739**	**556439**
**Yoruba**	**Corded_Ware_Latvia**	**LBK_EN**	**Loschbour_WHG**	**0.003892**	**6.216**	**103486**
**Yoruba**	**Poland_CWC**	**Loschbour_WHG**	**Yamnaya_Samara**	**−0.001771**	**−4.898**	**566961**
Yoruba	Corded_Ware_Estonia	Loschbour_WHG	Yamnaya_Samara	0.000182	0.555	554968
Yoruba	Corded_Ware_Germany	Loschbour_WHG	Yamnaya_Samara	0.00081	2.336	561626
Yoruba	Corded_Ware_Latvia	Loschbour_WHG	Yamnaya_Samara	0.000986	1.553	103225
**Yoruba**	**Poland_CWC**	**Loschbour_WHG**	**Afanasievo**	**−0.001423**	**−3.520**	**556752**
Yoruba	Corded_Ware_Germany	Loschbour_WHG	Afanasievo	0.000143	0.363	550919
Yoruba	Corded_Ware_Estonia	Loschbour_WHG	Afanasievo	0.000251	0.643	545651
Yoruba	Corded_Ware_Latvia	Loschbour_WHG	Afanasievo	0.001919	2.355	101461
**Yoruba**	**Poland_CWC**	**Loschbour_WHG**	**Corded_Ware_Estonia**	**−0.002257**	**−6.347**	**559019**
**Yoruba**	**Poland_CWC**	**Loschbour_WHG**	**Corded_Ware_Germany**	**−0.001526**	**−4.009**	**564772**
Yoruba	Poland_CWC	Loschbour_WHG	Corded_Ware_Latvia	−0.00175	−2.859	103966

### Mitochondrial DNA and Y-chromosome haplogroups

The analysis of the respective mtDNA and Y-chromosome haplogroups of the 17 individuals (Supplementary Tables S1–3) are in accord with the patterns observed for the nuclear genome-wide data. Most mtDNA lineages found are characteristic of the early Neolithic farmers in south-eastern and central Europe of the Starčevo-Kőrös-Criş and LBK cultures. Haplogroups N1a, T2, J, K, and V, which are found in the Neolithic BKG, TRB, GAC and Early Bronze Age samples, are part of the mitochondrial ‘Neolithic package’ (which also includes haplogroups HV, V, and W) that was introduced to Europe with farmers migrating from Anatolia at the onset of the Neolithic^17,31^.

A noteworthy proportion of Mesolithic haplogroup U5 is also found among the individuals of the current study. The proportion of haplogroup U5 already present in the earliest of the analysed Neolithic groups from the examined area differs from the expected pattern of diversity of mtDNA lineages based on a previous archaeological view and on the aDNA findings from the neighbouring regions which were settled by post-Linear farmers similar to BKG at that time^31,32^. A large proportion of Mesolithic haplogroups in late-Danubian farmers in Kuyavia was also shown in previous studies concerning BKG samples based on mtDNA only^33,34^, although these frequencies were derived on the basis of very small sample sizes.

We were able to derive the respective Y-chromosome haplogroup data for only four of the seven males in this study (one of which with a low confidence level) (Supplementary Tables S1 and S3). Results indicate that all three Neolithic individuals belonged to haplogroup I (I2a2a), and the fourth individual, dated to the Early Bronze Age, was assigned to haplogroup R (R1a).

The presence of haplogroup R1a is in accordance with evidence of a steppe migration into Europe from the East at the end of Neolithic^2,9^.

## Discussion

The analysis of a human time series from north-central Poland that spans from the Middle Neolithic to the Early Bronze Age (4300-1900 BCE) provides insights into the affinities and admixture patterns, and adaptive processes of farming populations in the Kuyavia region. A resurgence of WHG genetic component, with its most significant impact around the end of the Middle Neolithic period, has been identified in other studies^2,3,12,31^ and the same recurrent patterns of hunter-farmer admixture were identified in our study. Such results are similar to data for central Germany (Middle Elbe/Saale region)^2^, however in that case the resurgence of HG ancestry was found within the Funnel Beaker (TRB) complex dated to 4000-3000 BCE, while our data shows that admixture had occurred earlier and involved farmers representing post-Linear Pottery culture units. Consequently, the turning point in terms of hunter-farmer admixture was not between Danubian and “indigenous” Neolithic north-central European TRB and GAC cultures (traditional view presented in archaeological literature), but rather within the Danubian Neolithic tradition - between LBK and post-LBK cultural units. This may be in accordance with the demographic decline between these phases of the Danubian Neolithic seen not only in the Kuyavia region^27^, but in Central Europe in general^38,39^; it is possible that the close contacts with HG were a key for the success of post-LBK societies, which more effectively and permanently colonized the Polish lowlands.

A significant genetic influence of HG populations persisted in this region at least until the Eneolithic/Early Bronze Age period, when steppe migrants arrived to central Europe^2^. The presence of two outliers from the middle and late phases of the BKG in Kuyavia associated with typical Neolithic burial contexts (Supplementary Data S1) provides evidence that hunter-farmer contacts were not restricted to the final period of this culture and were marked by various episodes of interaction between two societies with distinct cultural and subsistence differences. The identification of both mitochondrial and Y-chromosome haplogroup lineages of Mesolithic provenance (U5 and I, respectively) in the BKG support the theory that both male and female hunter-gatherers became part of these Neolithic agricultural societies, as has been reported for similar cases from the Carpathian Basin^1^, and the Balkans^12,16^. The identification of an individual with WHG affinity, dated to ca. 4300 BCE, in a Middle Neolithic context within a BKG settlement, provides direct evidence for the regional existence of HG enclaves that persisted and coexisted at least for over 1000 years, from the arrival of the LBK farmers ca. 5400 BCE until ca. 4300 BCE, in proximity with Neolithic settlements, but without admixing with their inhabitants.

The analysis of two Late Neolithic cultures, the GAC and CWC, shows that steppe ancestry was present only among the CWC individuals analysed, and that the single GAC individual had more WHG ancestry than previous local Neolithic individuals. Similarly, an absence of steppe and high WHG ancestries in 5 GAC individuals from Kierzkowo, Poland, and 3 from Ilatka, Ukraine, was recently reported^12,15^. We note, however, that since the GAC individuals analysed both in^12,15^ and here are from the early parts of the GAC period, we cannot exclude the possibility of a steppe component in later GAC populations. The CWC’s affinity to WHG, however, contrasts with results from published CWC individuals that identified steppe ancestry related to Yamnaya as the major contributor to the CWC genomes^2,9^, while here we report also substantial contributions from WHG that could relate to the late persistence of pockets of WHG populations, as supported by the admixture results of N42 and the finding of the 4300-year-old N22 HG individual. These results agree with archaeological theories that suggest that the CWC interaction with incoming steppe cultures was complex and that it varied by region^40^.

New insights into Late Neolithic population dynamics suggest complex and variable events of admixture between local Kuyavia groups, who experienced a progressive resurgence of hunter-gatherer ancestry over a period of 2400 years. As in other areas of eastern Europe, at the end of the Neolithic period the arriving steppe migrants admixed with local populations but here with a smaller genetic footprint. Future studies on additional time transects from other regions in northern and central Europe are required in order to develop a more comprehensive pan-regional perspective on the genetic influence of Neolithic populations and the complex nature of hunter-farmer contact.

## Materials and Methods

### Bone Sampling

The osseous labyrinth (inner ear area) of each petrous bone was identified, isolated, and then ground to a fine powder^41^. This procedure was conducted in sterile and dedicated ancient DNA sample preparation facilities at University College Dublin and at the University of Lodz and all standard precautions were taken to avoid sample contamination.

### Ancient DNA Extraction

A first subset of 28 samples was analysed in University College Dublin with the purpose of screening for overall DNA quality and complexity from this geographical area and archaeological sites. A second set of 10 new samples was prepared in the University of Lodz. All extractions followed the same protocol, extracting approximately 50 mg of bone powder per sample^42^, and took place in dedicated ancient DNA labs in Dublin and Lodz, in adherence with strict anti-contamination protocols.

### Ancient DNA Library Preparation

We divided the DNA extracts of all samples into two portions of 12.5 uL. One portion was converted into libraries without any type of uracil-DNA glycosylase (UDG) treatment in order to identify the molecular damage patterns typical of ancient DNA molecules. The other portion was used for UDG-treated libraries once the screening stage was completed. For the non UDG-treated libraries, we used a modified version of ^43^ as outlined in^1^, where blunt end repair was performed using NEBNext End-Repair (New England Biolabs Inc.) and Bst was inactivated by heat (20 minutes at 80 °C). For the creation of full UDG-treated libraries, we followed^44^.

Libraries were quantified using a DNA-1000 chip on an Agilent 2100 Bioanalyser platform, and a Qubit 2.0 Fluorometer (Thermo Fischer Scientific), prior to pooling.

### Next-Generation Sequencing

The samples allocated for screening at UCD were sequenced at the UCD Conway Institute of Biomolecular and Biomedical Research, on an Illumina MiSeq platform, using 65 base pair (bp) and single-end sequencing. All final, UDG-treated, and the remaining 10 samples for screening, were sequenced on an Illumina NextSeq platform at the University of Lodz, using 100 bp single-end sequencing.

### Sequencing Data Processing

An ancient DNA bioinformatics pipeline was applied for processing of raw data. Cutadapt v1.5^45^ was used to trim adapter sequences with minimum overlap set to 1 (−O 1) and minimum length to 17 bp (−m 17). Reads were aligned to the hg19 build of the human genome using the Burrows-Wheeler Aligner^46^ with disabled seed (−l 1000) and with a minimum QC quality filter of 30. Duplicated sequences were removed using samtools v0.1.19-96b5f2294a^47^. General sequencing information is presented in Supplementary Table S1.

### Ancient DNA Authenticity

We selected 17 samples for deeper sequencing based on endogenous DNA yields and authenticity assessment, in order to obtain a coverage of the archaeological cultures in study that would allow us to answer our research questions. To assess DNA authenticity, we used the mapDamage2 tool^48^ to verify deamination patterns and frequencies and analysed length distributions on the samples from the screening steps. The 17 deep sequenced samples showed characteristic deamination patterns, with frequencies of substitution ranging from 0.13 to 0.34 on the 5′ end (C > T), and from 0.08 to 0.30 on the 3′ end (G > A) (Supplementary Fig. S2). The average read length for these samples on the screening run was between 45 and 52 bp (Supplementary Table S1) and between 46 and 65 bp for the deep sequenced samples (Supplementary Table S1). These patterns and values are all within the expected ranges for aDNA, where the molecules are of sizes shorter than 100 bp and present recognizable patterns of damage at both the 5′ end 3′ ends. Mitochondrial contamination of the samples was estimated to vary between 0 and 3%, by assessing mitochondrial DNA mutations in the individuals that are very rare in present-day humans, as in^49^. In order to ensure the absence of bias and fake clusters in our results induced by DNA damage, we repeated a subset of the same tests relying on transversions only. These included PCA (Supplementary Fig. S3), ADMIXTURE (Supplementary Fig. S3) and *f*_3_-statistics (Supplementary Table S9). The results were consistent between the analysis conducted on all loci and on the dataset that included only transversions.

### Molecular Sex and Uniparental Haplogroup Assessment

We applied the method of ^50^ for sex determination and the results showed with high confidence that, out of the 17 individuals included in this study, 10 are females and 7 are males. We then used published software to assess their uniparental haplogroups. For mitochondrial DNA we used Phy-Mer, a software that uses a reference-independent k-mer based approach^51^ and for Y chromosome we used YFitter v.0.2^52^. Results are presented in Supplementary Tables S1–3.

### Genotyping and Dataset Curation

We called single nucleotide polymorphisms (SNPs) with the Genome Analyzer Tool Kit’s (GATK) Pileup tool for the 594,924 autosomal positions present in the Affymetrix’s Human Origins dataset, described in^53^. For sites with coverage above 1x, we randomly selected an allele with quality above or equal to Q30. All SNPs from our samples and from^7,8^ were converted to pseudo-haploid calls by duplicating the randomly selected allele for that position. All these samples were merged with the dataset from^53^ using *mergeit* v.2450 from AdmixTools.

### Principal Component Analysis

Principal component analysis (PCA) was performed using the smartpca software (v.13050) from Eigensoft 5.0.1^54^. We included only a subset of 646 European, West Asian, and Near Eastern samples comprising 46 modern populations from the broader Human Origins dataset. All ancient samples from the dataset described on the previous section were projected onto these first two principal components by using the options *lsqproject:YES* and *shrinkmode:YES*.

### Population Structure

Population structure was determined using unsupervised ADMIXTURE v.1.2.3^55^. As suggested by the authors, the dataset was pruned for linkage disequilibrium with the option–indep-pairwise 200 25 0.4 using PLINK. This left us with 305255 SNPs for the analysis. Each K value from the range 2 to 14 (representing the number of ancestral populations) was repeated 5 times with random seeds. Cross validation errors (Supplementary Fig. S4) started to plateau on the lowest values at K = 10, and we chose the run that maximized the western hunter-gatherer component and showed clear distinction between that, the Early Neolithic, and the eastern steppe, present at K = 10 (Fig. 2). Modern populations are shown in Supplementary Fig. S5 (K = 10).

### *f*_*3*_ and *f*_*4*_-statistics

We used the package ADMIXTOOLS to perform outgroup-*f*_3_ and *f*_4_-statistics^56^. Due to the presence of outliers in some of the periods analysed, we performed outgroup-*f*_3_ of the form *f*_3_(Yoruba; X, Y) on each individual (Supplementary Fig. S6) and separately on each cultural set of individuals, with the outliers as independent individuals - BKG, TRB, GAC, CWC, and EBA (Fig. 3) to assess the level of shared genetic drift between X and Y after diverging from an outgroup population. Here X represents one of the Polish individuals/cultures, whereas Y is one of the remaining populations in the dataset, both modern and ancient, using Yoruba as outgroup.

We applied *f*_4_-statistics to test and confirm hypothesis revealed by previous analysis (Supplementary Table S4). The tests of the form *f*_4_(Yoruba, X; Y, Z) had X as the cultures/individuals, Y as the first population in the dataset to test against, and Z as the second with Yoruba as outgroup. The parameter *f*4*mode: YES* was used for these tests.

### *qpAdm* Analysis

We estimated admixture coefficients and modelled all ancient Polish group using *qpAdm* from ADMIXTOOLS^56^. This package allows to investigate if a target population or individual can be modelled as a result of admixture between two or more test populations. We followed the approach used in^53^, using the O9 outgroup list as a base, and when testing for the significance of lower rank models including the dropped population in the outgroup list to test for robustness of the results. Models were only accepted if both tests, with and without the dropped population as an outgroup, produced p > 0.05. In a few cases when the highest model was the only statistically significant, we added an extra outgroup to further test robustness (Supplementary Table S5).

### Relatedness Analysis

Relatedness analysis was performed on the four Corded Ware samples, that either archaeologically or during the analysis showed possible familial relationships (Supplementary Fig. S1). We used the method from^57^ (Supplementary Table S6).

## Electronic supplementary material

Supplementary Information

Supplementary Tables

## Data Availability

Raw sequencing data for all 17 ancient individuals is available at the European Nucleotide Archive and the National Center for Biotechnology Information with the accession number PRJNA318237.
