# Supplementary material for: A genomic Neolithic time transect of hunter-farmer admixture in central Poland
Source: Sci Rep. 2018 Oct 5;8:14879. doi: 10.1038/s41598-018-33067-w (PMC6173765; doi:10.1038/s41598-018-33067-w)
Supplement: Supplementary file 1 — Supplementary Information [file 41598_2018_33067_MOESM1_ESM.pdf]

A genomic Neolithic time transect of hunter-farmer admixture in central Poland

D. M. Fernandes, D. Strapagiel, P. Borówka, B. Marciniak, E. Żądzińska, K. Sirak, V. Siska, R. Grygiel, J. Carlsson, A. Manica, W. Lorkiewicz, R. Pinhasi

Supplementary Information

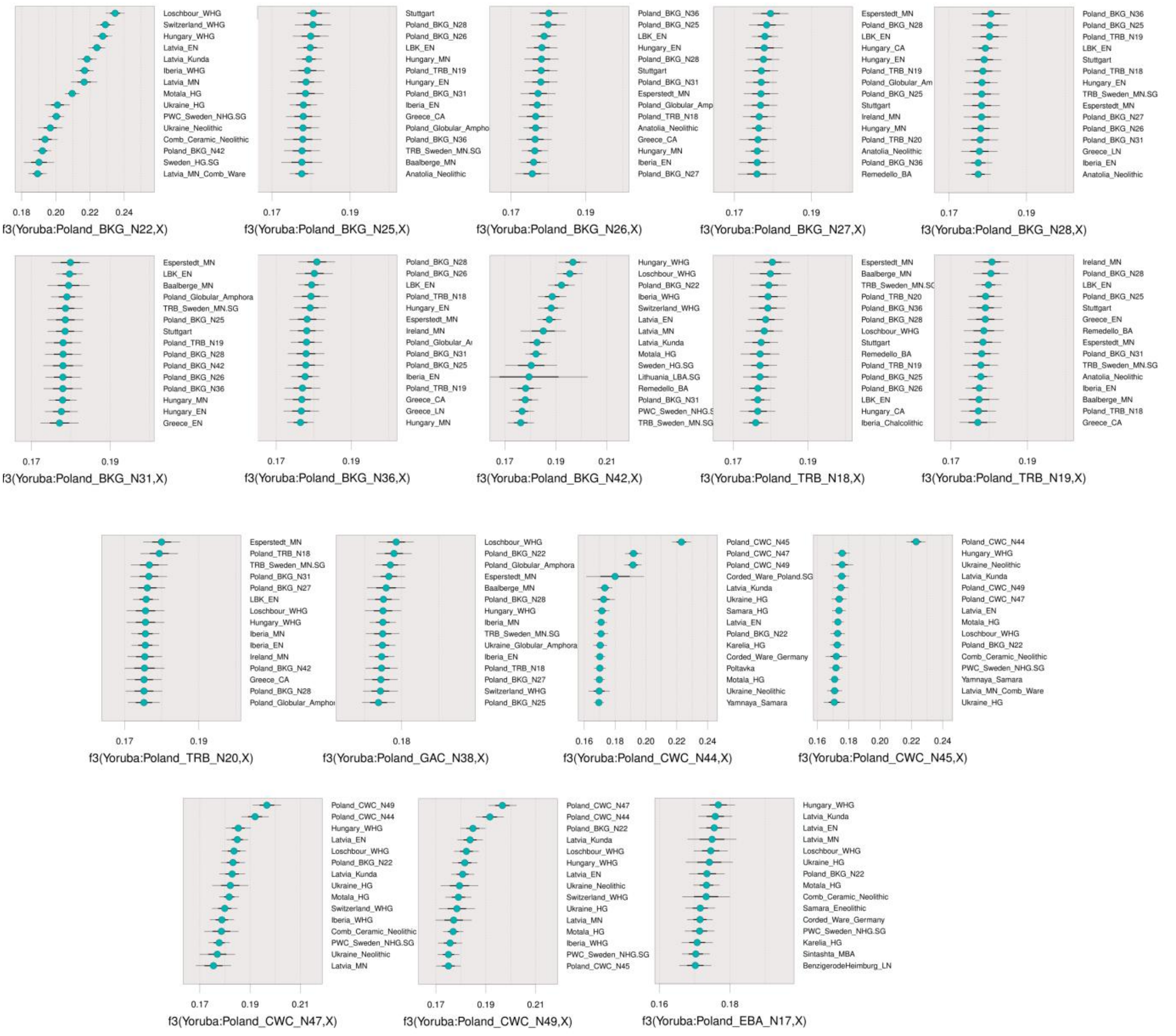

Supplementary Figure S1. Outgroup f_3 results for all 17 newly analysed individuals. Top 15 results shown

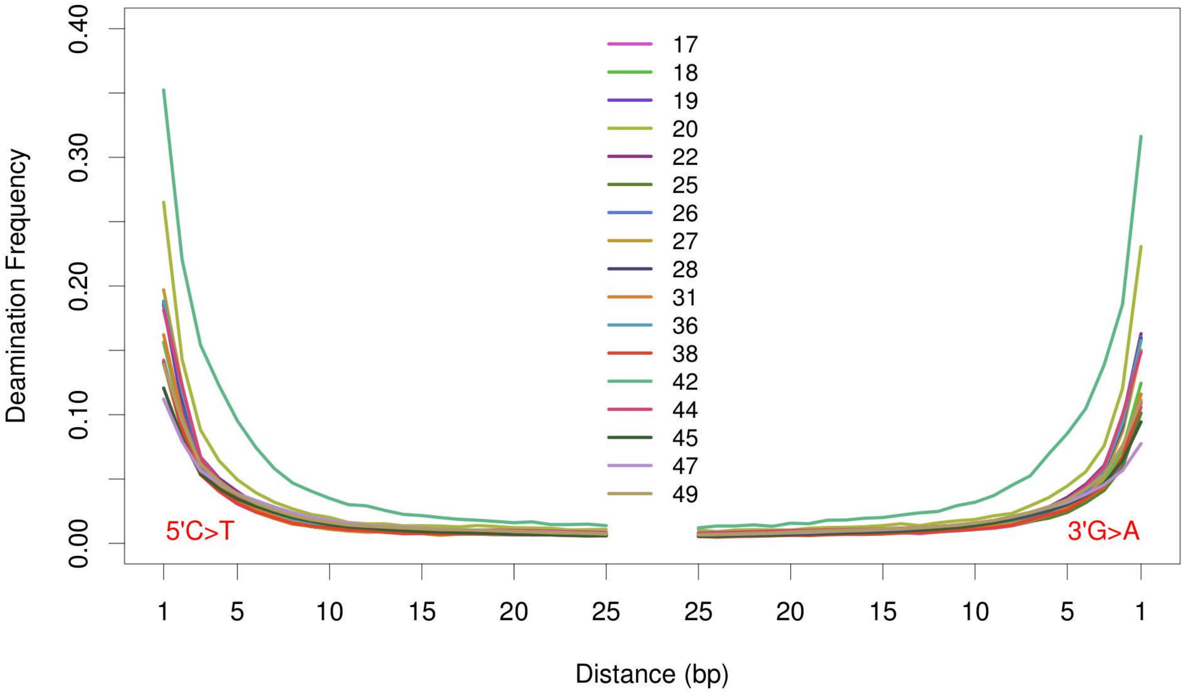

Supplementary Figure S2. Deamination frequencies for the 17 deep sequenced samples with sample number on the centre.

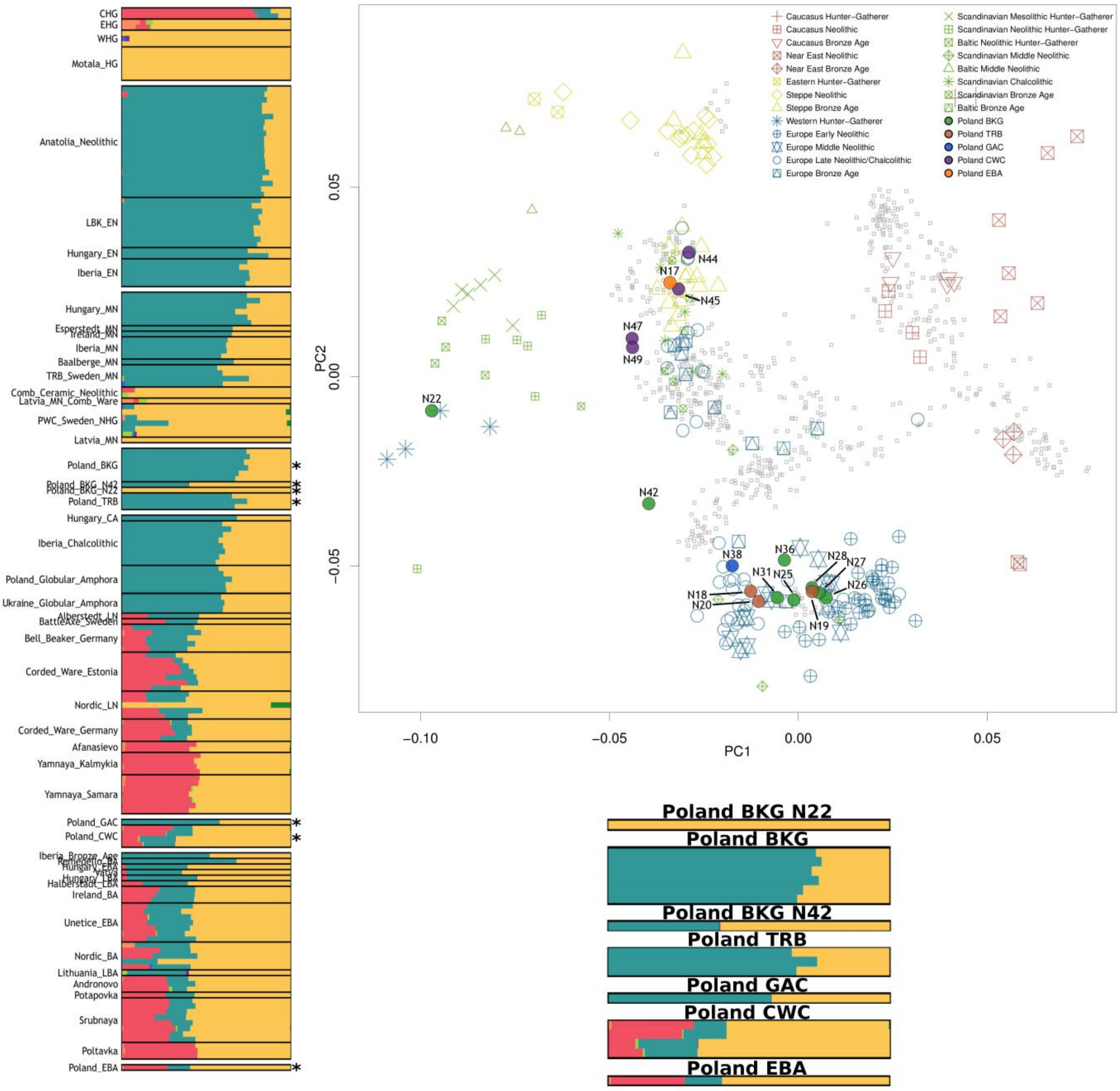

Supplementary Figure S3. Principal component analysis and ADMIXTURE results (K=10) for the dataset on transversion SNPs only.

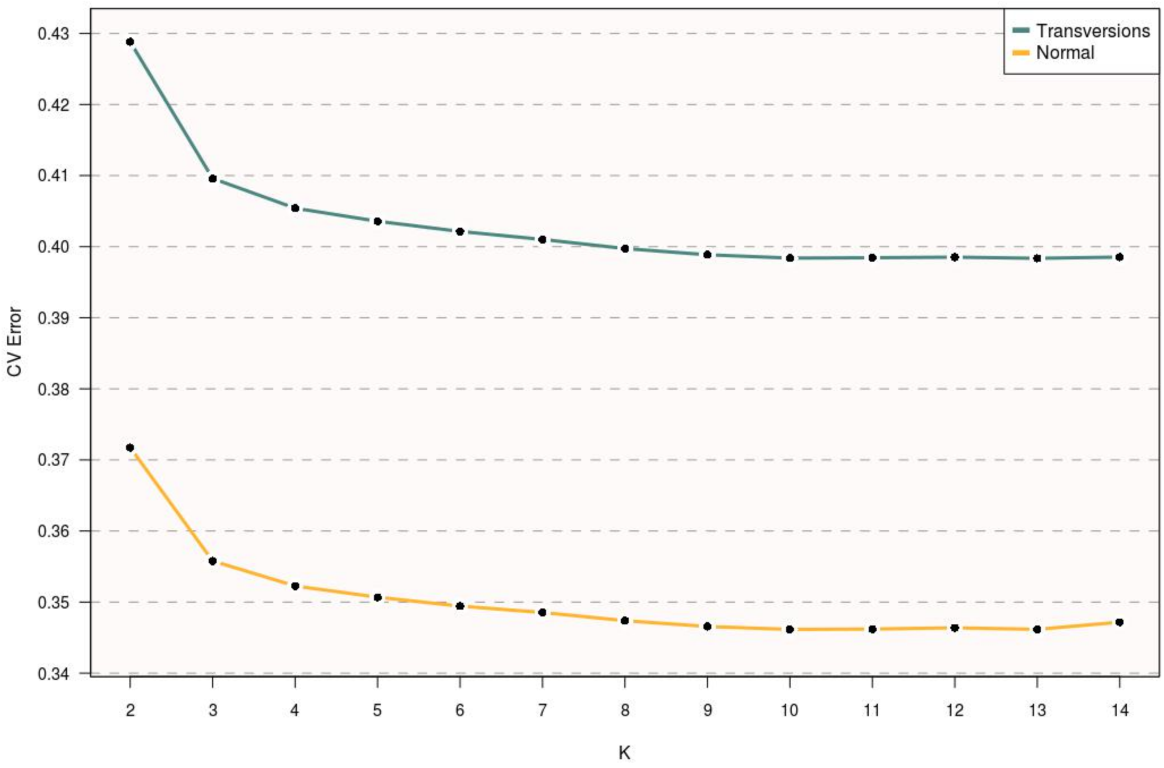

Supplementary Figure S4. Cross-validation errors for ADMIXTURE analysis for K (number of ancestral populations) values of 2 to 14.

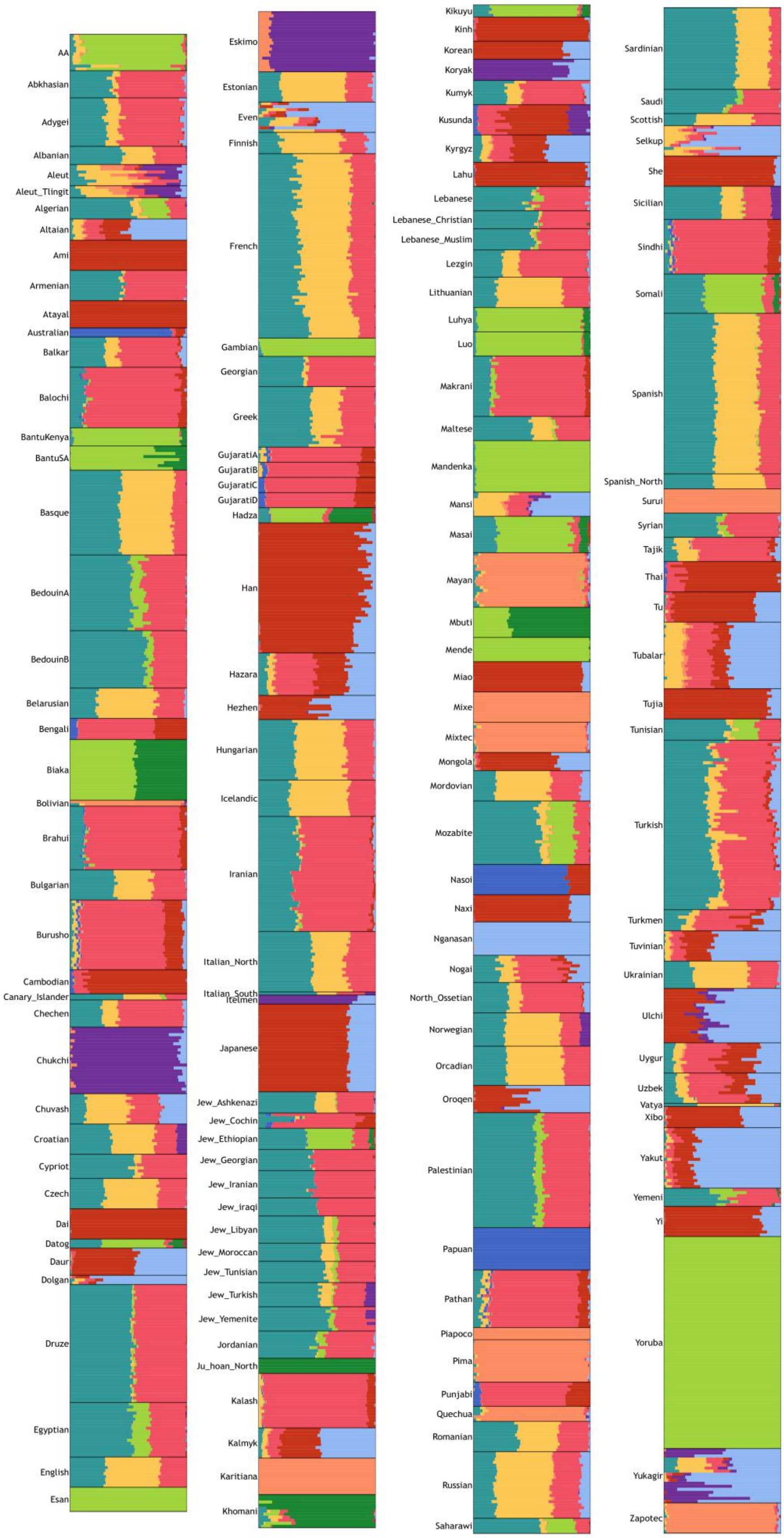

Supplementary Figure S5. ADMIXTURE results for modern populations for K=10, ordered alphabetically.

Supplementary Data S1. Archaeological Information and Contexts

The skeletons included in this study come from eight archaeological sites located within a radius of approximately 7 km around Brześć Kujawski in southern Kuyavia. All sites were excavated by the Museum of Archaeology and Ethnography in Łódź, and the unearthed skeletons were entrusted to the Department of Anthropology, University of Łódź, for anthropological and genetic analysis.

Skeletons attributed to the Brześć Kujawski Group of the Lengyel Culture (BKG) come from four sites: Brześć Kujawski site 3, Osłonki site 1, and Konary sites 1 and 1a, all of which consist of remains of settlements with groups of burials located within their perimeter. The BKG is divided into three phases: Early (ca. 4600-4500 BCE), Middle or Classic (ca. 4500-4300 BCE), and Late (ca. 4300-400 BCE), with the individuals from this study representing the Middle and Late phases. The deceased were inhumed in single (and sometimes double or even triple) graves in a contracted position on the right or left side, depending on the sex (Supplementary Figure S6)[1]. Also some elements of grave goods were sex-specific. As many as 180 skeletons were uncovered in the discussed region from BKG sites, including the four mentioned above as well as another five; they comprise the largest prehistoric and culturally homogeneous Neolithic skeletal series from the area of present-day Poland.

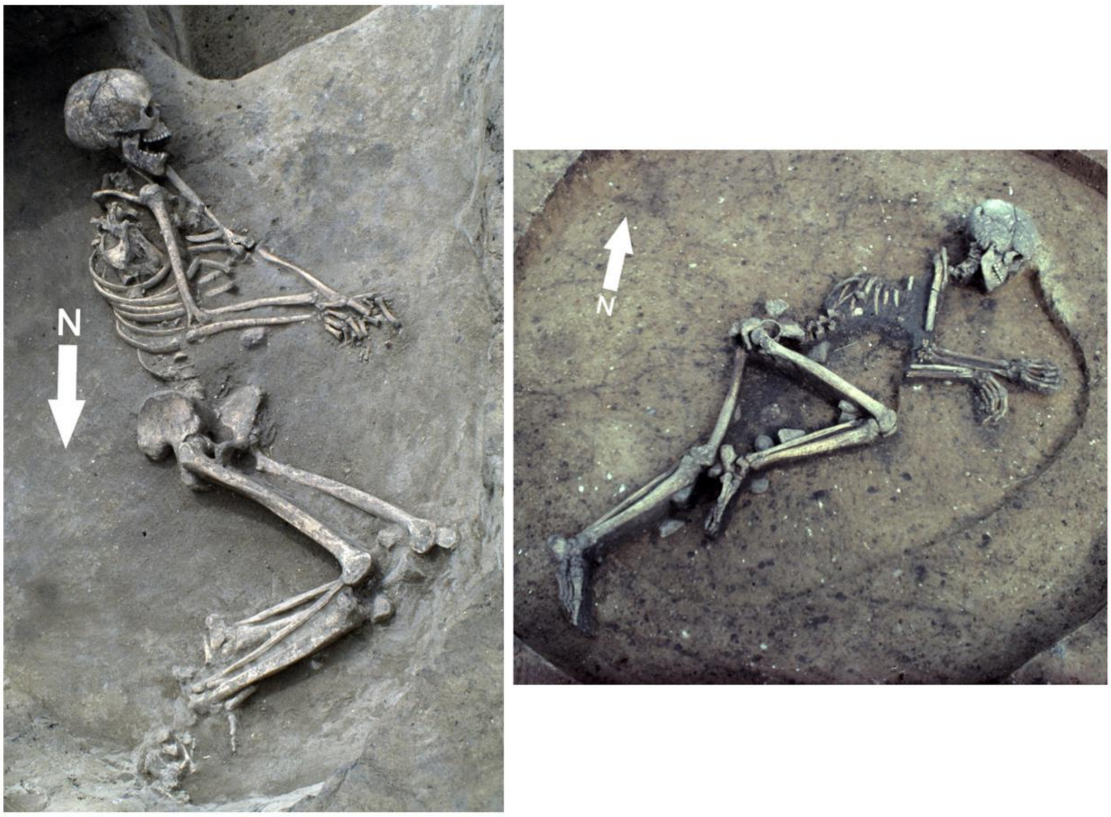

Supplementary Figure S6. Left: Burial of the Brześć Kujawski group of the Lengyel culture from the Osłonki site 1 (individual N27). Right: Burial of the Brześć Kujawski group of the Lengyel culture from the Brześć Kujawski site 3 (individual N22).

Among the examined BKG burials, of particular note is grave 70 from site BK3 (individual N22), which was discovered in the part of the site containing the relics of a Linear Pottery Culture (LBK) settlement (Fig S1). In the absence of archaeological artifacts, the grave was initially associated with LBK, but C^{14} dating (Supplementary Table S1) revealed that it was a BKG burial atypically situated beyond the perimeter of the relics of that culture present at that site (including other BKG graves). The skeleton belonged to an individual aged 15–20 years old and of morphologically undeterminable sex.

The other studied BKG burials were located within the remains of three coeval settlements: a large, central settlement in Osłonki and two satellite settlements in Konary (sites 1 and 1a). The morphological age and sex determinations for the studied individuals are as follows: N31 – female, approx. 30–35 years old; N27 – male, 40–50 years old; N25 – undetermined sex, 17–20 years old; N42 – female, 30–40 years old; N28 – male, approx. 20–25 years old; N26 – male, 45–55 years old; N36 – female, 20–30 years old.

Skeletons attributed to the Funnel Beaker Culture (TRB) come from Pikutkowo sites 5 and 6. TRB burials differ significantly from those of BKG: they are typically single, constructed of stones arranged in the form of a chest, with the deceased buried in a straight supine position or in a slightly contracted position on the side[2]. The morphological sex and age determinations for the studied individuals are as follows: N18 – undetermined sex, 11–15 years old; N19 – female, above 50 years old; N20 – male, 25–35 years old.

The Globular Amphora culture (GAC) skeleton was collected from a double burial discovered at site 4 in Brześć Kujawski (BK4 site) and was a secondary object within the remains of a BKG settlement. The burial, which was surrounded by large stones, contained the skeleton of a 20–30-year-old female (individual N38 included in the study), as well as a male aged over 50 years (not included)[3]. The BK4 site is an example of multicultural settlement sequences, which were quite frequent in the region of Kujawy.

All the studied skeletons attributable to the Corded Ware culture (CWC) come from one multiple burial discovered at Pikutkowo site 6, containing the remains of 14 individuals, both adults and children[4]. This site is also multicultural; apart from the aforementioned CWC grave, it consists of the remains of BKG and TRB settlements and burial grounds. The present study involved four skeletons: an adult female (N44) as well as three children aged approximately 13–15 years (N45), 5–7 years (N49), and approximately 5–6 years (N47).

The skeleton from the Early Bronze Age (individual N17) comes from a damaged burial discovered at the Gustorzyn site. Despite the absence of archaeological artifacts, the absolute dating of the skeleton suggests that the burial must have belonged either to the Iwno culture or to the early Trzciniec culture. Morphologically, the skeleton was of a 40–50-year-old male.

References

1 Grygiel, R. *The Neolithic and Early Bronze Age in the Brześć Kujawski and Osłonki region. Vol. 1. Early Neolithic. Linear Pottery culture*. (Fundacja Badań Archeologicznych Imienia Profesora Konrada Jażdżewskiego and Muzeum Archeologiczne i Etnograficzne w Łodzi, 2004).

2 Grygiel, R. *The Neolithic and Early Bronze Age in the Brześć Kujawski and Osłonki region. Vol. III. Middle and Late Neolithic. The Funnel Beaker culture*. (Fundacja Badań Archeologicznych Imienia Konrada Jażdżewskiego, Muzeum Archeologiczne i Etnograficzne w Łodzi, 2016).

3 Jażdżewski, K. Cmentarzyska kultury wstęgowej i związane z nimi ślady osadnictwa w Brześciu Kujawskim. *Wiadomości Archeologiczne* **15,** 1–105 (1938).

4 Niesiołowska, E. Materiały neolityczne ze stanowiska 6 w Pikutkowie, pow. Włocławek. *Prace i Materiały Muzeum Archeologicznego i Etnograficznego w Łodzi* **14,** 79–144 (1967).
